# Assessment of genetic diversity in Brazilian barley using SSR markers

**DOI:** 10.1590/1678-4685-GMB-2015-0148

**Published:** 2016

**Authors:** Jéssica Rosset Ferreira, Jorge Fernando Pereira, Caroline Turchetto, Euclydes Minella, Luciano Consoli, Carla Andréa Delatorre

**Affiliations:** 1 Departamento de Plantas de Lavoura, Faculdade de Agronomia, Universidade Federal do Rio Grande do Sul, Porto Alegre, RS, Brazil; 2 Embrapa Trigo, Passo Fundo, RS, Brazil

**Keywords:** Domesticated barley, genetic diversity, *Hordeum vulgare* ssp. vulgare, *Hordeum vulgare* ssp. spontaneum, microsatellite markers

## Abstract

Barley is a major cereal grown widely and used in several food products, beverage production and animal fodder. Genetic diversity is a key component in breeding programs. We have analyzed the genetic diversity of barley accessions using microsatellite markers. The accessions were composed of wild and domesticated barley representing genotypes from six countries and three breeding programs in Brazil. A total of 280 alleles were detected, 36 unique to Brazilian barley. The marker Bmag120 showed the greatest polymorphism information content (PIC), with the highest mean value found on chromosome three, and the lowest on chromosomes four and six. The wild accessions presented the highest diversity followed by the foreign genotypes. Genetic analysis was performed using Principal Coordinates Analysis, UPGMA clustering, and Bayesian clustering analysis implemented in Structure. All results obtained by the different methods were similar. Loss of genetic diversity has occurred in Brazilian genotypes. The number of alleles detected in genotypes released in 1980s was higher, whereas most of the cultivars released thereafter showed lower PIC and clustered in separate subgroups from the older cultivars. The use of a more diverse panel of genotypes should be considered in order to exploit novel alleles in Brazilian barley breeding programs.

## Introduction

Barley (*Hordeum vulgare*) was one of the first cereal species domesticated by humans and, since then, it is cultivated in several environments (Ladizinsky 1998; Feuillet *et al.*, 2007). It is considered one of the best adapted cereals due to its tolerance to salinity, low temperatures and lower water demand (Baik and Ullrich, 2008;Goyal and Ahmed, 2012), although it is highly sensitive to aluminum (Bian *et al.*, 2013). A broad range of end use, such as human consumption, malt in brewing and distilling industry and animal feeding, makes barley one of the most important cereal crops in the world ranking as the fourth most produced cereal after wheat, maize, and rice.

Cultivated barley (*Hordeum vulgare* ssp. *vulgare*) and its wild progenitor (*Hordeum vulgare* ssp.*spontaneum*) belong to the barley primary gene pool (Minella, 2005). Greater allelic diversity has been detected in wild than in cultivated barley (Saghai Maroof *et al.*, 1994; Ledovskoy *et al.*, 2010; Nandha and Singh, 2014).

The wild progenitor is of great interest for crop breeding because it carries a rich source of genes, enabling the adaptation to different biotic and abiotic stresses (Nevo *et al.*, 2012). Moreover, these species preserved their intercrossing ability. The genetic diversity found in cultivated barley varies among different studies, some of them reporting high genetic diversity (Baik and Ullrich, 2008; Yahiaoui *et al.*, 2008; Khodayari *et al.*, 2012). Previous studies also showed different levels of genetic diversity in accessions of different geographic origins. For instance, genotypes from Europe showed lower genetic diversity compared to those of other continents (Malysheva-Otto *et al.*, 2006).

In the last years, Brazil imported around 350 thousand tons yearly to supply the domestic needs, and 75% of the barley has been destined to produce malt for beer brewing (de Mori and Minella, 2012). Area expansion, and consequently production increase, would require the development of cultivars adapted to the diverse Brazilian environments. Hence, knowledge about the allelic composition of materials may facilitate the parental selection for breeding to specific environments. However, up to now, few genetic variability studies have been conducted for Brazilian barley. SDS-PAGE analysis was employed to analyze the hordein polypeptide patterns, which revealed a surprisingly extensive intravarietal polymorphism for Brazilian barley varieties (Echart-Almeida and CavalliMolina, 2000). The intravarietal variability was also detected by RAPD and isoenzymes, although in lower frequency (Selbach and Cavalli-Molina, 2000; Maris AF, 1992, Bachelor's thesis, Universidade Federal do Rio Grande do Sul, Porto Alegre, RS). However, a great genetic similarity was found among cultivars (Echart-Almeida and Cavalli-Molina, 2000; Kroth *et al.*, 2005; Maris AF, 1992, Bachelor's thesis, Universidade Federal do Rio Grande do Sul, Porto Alegre, RS). Another good option for cultivar characterization is the use of microsatellite markers (SSR) that possess high levels of polymorphism, are multiallelic and spread throughout the genome (Gupta *et al.*, 1996; Oliveira *et al.*, 2006). Although SSR markers have been used to characterize barley from different countries (Saghai Maroof *et al.*, 1994; Karakousis *et al.*, 2003; Baik and Ullrich, 2008; Yahiaoui*et al.*, 2008; Ledovskoy *et al.*, 2010; Khodayari *et al.*, 2012; Nandha and Singh, 2014), no studies evaluating the molecular diversity of Brazilian barley using this kind of markers have been conducted.

The objectives of the present study were to describe the molecular diversity of Brazilian barley, as well as to compare that diversity with the one found for foreign and wild genotypes. The aim was to detect new genetic variability and thus to assist the breeding programs in the development of new cultivars.

## Material and Methods

### Plant material and genomic DNA isolation

Seeds of 64 barley accessions, provided by the Active Germplasm Bank of CNPT-Embrapa, Brazil, were cultivated under controlled conditions. These accessions represent two- and six-row materials including six wild (*Hordeum vulgare* ssp. *spontaneum*) genotypes, 35 cultivars and 16 breeding lines developed by different breeding programs in Brazil, and seven cultivars from other countries (Table 1). The Brazilian accessions used here have spring growth habit and are representative of the germplasm cultivated in Brazil in the last four decades. Because we were interested in evaluating the genetic variability among the different Brazilian breeding programs (development institution), the wild and domesticated accessions, the breeding lines and cultivars, and among the Brazilian and foreign genotypes, we grouped the 64 barley accessions in seven sets according to development institution, country, and type as detailed in Table 2.

**Table 1:** Barley genotypes used in this study.

Genotype	Row	Institution	Country	Year	Pedigree
Alpha	2	Foreign	USA	**-**	'Manchuria'/'Champion of Vermont'
Antarctica 01	2	Cervejaria Antarctica Paulista	Brazil	1960's	Selection of Breun Volla
Antarctica 04	2	Cervejaria Antarctica Paulista	Brazil	1999	Alpha1959/Union
Antarctica 05	2	Cervejaria Antarctica Paulista	Brazil	1999	Unkown
BRS 180	6	CNPT/Embrapa	Brazil	1999	73Ab2199/Karla
BRS 195	2	CNPT/Embrapa	Brazil	2001	Defra/BR 2
BRS 224	2	CNPT/Embrapa	Brazil	2002	Embrapa 43/PFC 9114
BRS 225	2	CNPT/Embrapa	Brazil	2002	PFC 9103/Defra
BRS Brau	2	CNPT/Embrapa	Brazil	2009	MN 698/3/BRS 195//Schooner/Embrapa 129
BRS Cauê	2	CNPT/Embrapa	Brazil	2008	BRS Borema/BRS 195
BRS Elis	2	CNPT/Embrapa	Brazil	2008	BRS 195/Scarlett
BRS Greta	2	CNPT/Embrapa	Brazil	2006	Krona/PFC 9219//PFC 9204
BRS Itanema	2	CNPT/Embrapa	Brazil	2013	BRS 195//PFC8590/PFC9205
BRS Lagoa	2	CNPT/Embrapa	Brazil	2005	PFC 9215/PFC 9288
BRS Marciana	2	CNPT/Embrapa	Brazil	2005	PFC 9240/PFC 9211
BRS Mariana	2	CNPT/Embrapa	Brazil	2005	PFC 88137/PFC 8905//PFC 9205
BRS Sampa	2	CNPT/Embrapa	Brazil	2008	BRS 195//PFC 8590/PFC 9205
BRS Suábia	2	CNPT/Embrapa	Brazil	2006	BRS 195/MN 698
Cevada BR1	2	CNPT/Embrapa	Brazil	1987	Selection of W 5586 =Binder/Opal//Balder/kenia/3/Alpha
Cevada BR2	2	CNPT/Embrapa	Brazil	1989	FM 424/TR 206
Dayton	6	Foreign	USA	1955	Composite cross X (CI 6625) selection
Embrapa 127	2	CNPT/Embrapa	Brazil	2000	BR2/Alexis
Embrapa 128	2	CNPT/Embrapa	Brazil	1999	LM 844/PFC 84148//BR2
Embrapa 129	2	CNPT/Embrapa	Brazil	1999	LM 844/MN610//Cevada BR 2
Embrapa 43	2	CNPT/Embrapa	Brazil	1999	IPB 194//C 2146/TR 208
FM 404	2	Maltaria Navegantes (Filial Maltaria)	Brazil	1970's	Selection from a cross probably involving Alpha
FM 424	2	Maltaria Navegantes (Filial Maltaria)	Brazil	1970's	Quinn/Malteria Heda//W 5746
FM 434	2	Maltaria Navegantes (Filial Maltaria)	Brazil	1970's	Quinn/Malteria Heda//FM 424
FM 437	2	Maltaria Navegantes (Filial Maltaria)	Brazil	1985	Alpha 1959/2*Mansholt Twerijge Zomergerst
FM 519	2	Maltaria Navegantes (Filial Maltaria)	Brazil	1985	Kr 1/Union//Volla/3/Kr 2/Volla//Wisa/4/Alpha
Golden Promise	2	Foreign	England	1966	Maythorpe Gamma-Ray Mutant
Hspo 584	2	-	-	-	-
Hspo PI 282590	2	-	Israel	-	-
Hspo PI 466338	2	-	Israel	-	-
Hspo PI 466394	2	-	Israel	-	-
Hspo PI 466396	2	-	-	-	-
Hspo PI 466381	2	-	Israel	-	-
IAC 74310	2	Instituto Agronômico de Campinas	Brazil	1974	Unkown
IPB 1219	2	International Plant Breeders	Brazil	1980	Vada/Zephyr
IPB 194	2	International Plant Breeders	Brazil	1980	Mazurka/Nackta
MN 599	2	Maltaria Navegantes	Brazil	1990	Ariana/Volla//FM 462
MN 6021	2	Maltaria Navegantes	Argentina	2012	Dominique/Quilmes Ayelen
MN 656	2	Maltaria Navegantes	Brazil	1993	SG 4279/FM 404//Bacco/Union/3/ FM 434
MN 684	2	Maltaria Navegantes	Brazil	2001	Antarctica 05/MN 577
MN 698	2	Maltaria Navegantes	Brazil	2001	MN 599/MN 635
MN 743	2	Maltaria Navegantes	Brazil	2004	MN 681/Gimpel
Murasakimochi	6	Foreign	Japan	-	-
Paraí-I	6	CNPT/Embrapa	Brazil	-	Unkown
PFC 7802	2	CNPT/Embrapa	Brazil	7802	Binder/Opal//Balder/Kenya
PFC 8115	2	CNPT/Embrapa	Brazil	1981	Volla*3/Wpgm 626-46-25
PFC 8153	2	CNPT/Embrapa	Brazil	1981	FM 424*2//Volla/Wpgm 626-46-25
PFC 8280	2	CNPT/Embrapa	Brazil	1982	Volla/C 2146
PFC 84148	2	CNPT/Embrapa	Brazil	1984	Volla*3/Wpgm 626-46-25//MAGNIF 131
PFC 8601	2	CNPT/Embrapa	Brazil	1986	Seleção em TR 207 em solo corrigido (1/8 SMP)
PFC 8610	2	CNPT/Embrapa	Brazil	1986	PFC 8144/Park
PFC 86125	2	CNPT/Embrapa	Brazil	1986	PFC 8153/IPB 194
PFC 8644	2	CNPT/Embrapa	Brazil	1986	Antarctica 05*3/Park
PFC 88209	6	CNPT/Embrapa	Brazil	1988	Selection of FM 70
PFC 88210	6	CNPT/Embrapa	Brazil	1988	Selection of FM 71
PFC 88211	6	CNPT/Embrapa	Brazil	1988	Selection of FM 80
PFC 88212	6	CNPT/Embrapa	Brazil	1988	Selection of FM 80
Quench	2	Foreign	England	-	Sebastian/Drum
Quest	6	Foreign	USA	-	MN Brite/Zhedar 1
Vacaria	6	CNPT/Embrapa	Brazil	-	Unkown

**Table 2:** Groups of genotypes separated according to institution, country and barley type.

	CNPT/Embrapa cultivars	Companhia Antarctica Paulista	Maltaria Navegantes	Breeding lines from other institutions^1^	Breeding lines from CNPT/Embrapa^2^	Foreign genotypes	Wild barley
Number of genotypes	22	3	10	3	13	7	6
Total of alleles	146	61	105	63	130	123	145
Alleles per locus	4.29	1.79	3.09	1.85	3.82	3.62	4.26
PIC	0.46	0.27	0.43	0.32	0.52	0.57	0.63

1 Lines from Instituto Agronômico de Campinas and International Plant Breeders.

2 Genotypes identified in Table 1 as "PFC".

Young leaves of the seedlings were collected, transferred to 2 mL Eppendorf tubes containing three stainless steel beads (2.3 mm diameter) and immediately frozen in liquid nitrogen. Tissue was powdered using a Mini-Beadbeater™ (Biospec Products) platform and total genomic DNA was extracted using a CTAB protocol (Doyle and Doyle, 1987). The DNA quality and quantity was estimated on 0.8% agarose gels. DNA was stored at −20 **°** C for further analyses.

### Microsatellite (SSR) amplification

Genomic DNA was amplified using 34 previously described SSR markers (Liu *et al.*, 1996; Röder *et al.*, 1998; Ramsay *et al.*, 2000; Varshney *et al.*, 2007;Soto-Cerda *et al.*, 2013) that are scattered throughout the barley genome. The details of the 34 SSR primers are available in Table S1.

PCR was conducted based on a three primer system, as described by Schuelke (2000), where one of the locus-specific primers (forward or reverse) was extended with a non-labeled M13-tail (TGTAAAACGAC GGCCAGT), and a M13 primer labeled with a fluorescent dye (FAM, NED, PET or VIC). The reaction mixture was prepared in a final volume of 10 μL containing 1x buffer (RBC Bioscience), 0.2 μM primer, 0.02 μM M13 tailed primer and 0.2 μM fluorescence-labeled M13 primer. Other reagents were optimized for each primer and added as mixA [1.5 mM MgCl_2_ (RBC Bioscience), 0.2 mM of each dNTP (Thermo Scientific), 0.5 U of *Taq* polymerase (RBC Bioscience)], mix C [2.5 mM MgCl_2_, 0.2 mM of each dNTP, 0.75 U of *Taq* polymerase], or mixD [2.5 mM MgCl_2_, 0.35 mM of each dNTP, 0.75 U of *Taq* polymerase]. Amplification was performed in a GenAmp^®^ PCR System 9700 (Applied Biosystems) with two programs set according to the melting temperatures of the primers (Table S1): TD60-50 (94 °C for 30 s, 60 °C for 30 s, and 72 °C for 30 s, followed by 10 cycles at decreasing annealing temperatures of 1 °C per cycle and then 30 cycles at 94 °C for 30 s, 50 °C for 30 s, 72 °C for 30 s), or TD60-55 (94 °C for 30 s, 60 °C for 30 s, and 72 °C for 30 s, followed by five cycles at decreasing annealing temperatures of 1°C per cycle and then 35 cycles at 94 °C for 30 s, 55 °C for 30 s, 72 °C for 30 s). After amplification, the reactions from up to four primer combinations containing different fluorescent dyes were multiplexed, diluted, mixed with Hi-Di formamide and GeneScan 500 LIZ size standard (Applied Biosystems), denatured and run on an ABI 3130xL Genetic Analyzer containing a 36 cm capillary array with POP7 polymer. The program GeneMapper v3.5 was used to determine allele sizes.

### Data analysis

FSTAT 2.9.3.2 software (Goudet, 2002) was used to evaluate summary statistics, such as the number of alleles per locus (*A*) and number of private alleles (*E*) for each locus and groups of accessions. Polymorphism information content (PIC) was estimated using the PowerMarker software (Liu and Muse, 2005) according to Anderson*et al.* (1993): PIC = 1 - ∑ **P** *_ij_* ^2^, where P*_ij_* is the frequency of the*i*th allele at the *j*th marker, to evaluate the diversity level of each SSR marker. In addition, we implemented analyses of molecular variance (AMOVA; Excoffier *et al.*, 1992) using ARLEQUIN 3.5.1.2 software (Excoffier and Lischer, 2010) among seven groups of accessions. Grouping of the accessions was established according to the institution that developed the cultivars or breeding lines (Table 1). The genetic diversity among Brazilian genotypes developed in different decades was estimated based on the number of alleles, frequency of alleles and PIC values.

To investigate the genetic similarity between accessions, we carried out a Principal Coordinates Analysis in GENALEX 6.4 software (Peakall and Smouse, 2006, 2012). A matrix of distance based on shared alleles among accessions was used to depict relationships among all accessions; the original data were bootstrapped 10,000 times using PowerMarker software (Liu and Muse, 2005). In addition, an Unweighted Pair Group Method with Arithmetic Mean (UPGMA) tree was constructed based on the matrix of shared microsatellite alleles among the 64 accessions calculated from 34 SSRs using the PowerMarker software (Liu and Muse, 2005). A Bayesian clustering analysis as implemented in STRUCTURE 2.3 software (Pritchard *et al.*, 2000) was performed to compare to UPGMA and PCoA results. This approach uses a Bayesian clustering analysis to assign individuals to clusters (K) without prior knowledge of their population affinities. The parameters were correlated to allele frequencies (Falush*et al.*, 2003) and no prior accession information was used. The number of groups (K) was evaluated from one to 20, with 10 independent runs per K value, to determine the maximum value of the posterior likelihood [lnP(D)] and the best value of K. Each run was performed using 2.5 10^5^ burn-in periods and 10^6^ Markov chain Monte Carlo (MCMC) repetitions after burn-in, and the convergence was checked. The optimal value of K was calculated using the maximum value of ΔK (Evanno *et al.*, 2005) implemented in STRUCTURE HARVESTER 0.6.93 (Earl and von Holdt, 2012). CLUMPP 1.1.2 software was used to summarize the results of the optimal K value on the basis of the average pairwise similarity of individual assignments across runs using Greedy's method and the G' statistic (Jakobsson and Rosenberg, 2007). DISTRUCT 1.1 software (Rosenberg, 2004) was used to visualize the STRUCTURE results after processing with the CLUMPP software.

## Results

### Genetic variation

We have evaluated 34 SSR loci in 64 barley accessions including two- and six-row genotypes as well as wild and domesticated barley. Although 11 genotypes from six countries are among the ones studied here, the majority of accessions represent genotypes developed in Brazil. The 34 markers were spread through the barley genome with at least four markers on each chromosome. We detected 280 alleles with an average of eight alleles per locus. The number of alleles per locus varied from one for markers XGWM6, HvLOX and Bmac0251 (monomorphic loci) to 18 for the marker Bmag0032 (Table 3). Sixteen markers detected at least 10 alleles and only eight amplified less than five alleles. For only one marker (Bmac0167), a null allele was observed. An association between the number of motif repeats and the number of alleles could be established. On average, loci containing 15 or more motif repeats generated 10 alleles per marker while the ones with less than 15 motif repeats showed five alleles per marker. Among the monomorphic loci, two contained less than 15 motif repeats while one (XGWM6) possessed the highest number (40 repeats) among the evaluated markers. The highest number of alleles was found in the 22 cultivars developed by CNPTEmbrapa (146 alleles averaging four alleles per locus) and in the six wild accessions (145 alleles averaging four alleles per locus). However, considering the number of accessions within each set, the foreign genotypes also generated a high number of alleles (Table 2).

**Table 3:** Repeat motif, number of alleles, allele size range, and polymorphism information content (PIC) of the 34 SSR markers used.

Marker	Repeat motif	Number of alleles	Allele size range	PIC
Bmac0090	(AC)20	10	207-247	0.75
Bmag0032	(AC)7T(CA)15(AT)9	18	211-319	0.78
Bmag211	(CT)16	6	193-207	0.54
HVM20	(GA)19	6	150-172	0.66
Bmag0125	(AG)19	10	136-162	0.77
Bmag0378	(AG)14	8	150-170	0.26
Bmag0749	(AG)11	4	180-186	0.62
HVM36	(GA)13	6	124-166	0.65
HVM54	(GA)14	7	166-182	0.58
Bmac0067	(AC)18	9	180-250	0.72
Bmag0013	(CT)21	12	147-183	0.81
Bmag225	(AG)26	10	155-185	0.81
HVM60	(AG)11(GA)14	10	101-133	0.77
Bmac0030	(AC)22	11	150-180	0.83
Bmac0310	(CT)11(AC)20	11	159-199	0.76
Bmag0353	(AG)21	11	105-145	0.69
EBmac0669	(AC)8	2	236-238	0.07
EBmag0781	(CT)21	4	162-182	0.26
HVM68	(GA)22	12	196-240	0.77
HvML03	(CTT)6	3	251-266	0.33
wms165	(GA)20	8	215-263	0.23
XGWM6	(GA)40	1	196	0.00
Bmac0113	(AT)7(AC)18	10	208-236	0.81
Bmac096	(AT)6(AC)16	11	180-212	0.77
Bmag0387	(AG)16	8	119-161	0.53
HvLOX	(AG)9	1	170	0.00
Bmac0316	(AC)19	14	142-200	0.66
Bmac251	(AC)12A(AC)13	1	161	0.00
Bmag0173	(CT)29	8	156-180	0.79
HVM65	(GA)10	3	139-143	0.30
Bmac0031	AC(28)	10	172-211	0.53
Bmac0167	AC(20)	10^1^	180-212	0.76
Bmag0120	(AG)15	17	230-290	0.86
Bmag0135	(AG)10GG(AG)12	8	133-179	0.79

1 Null allele was observed.

Private alleles were detected in all sets except from Companhia Antarctica Paulista (Table S2). In the wild accessions, 69 private alleles were detected in 28 different loci (Table S2). Among the foreign genotypes and the cultivars developed by CNPT-Embrapa we found 23 and 21 private alleles, respectively. Only one private allele was visualized in the breeding lines developed by other institutions. The Bmag0032 marker was the locus with the highest number of alleles and presented the highest number of private alleles (Table S2).

**Figure 1:**
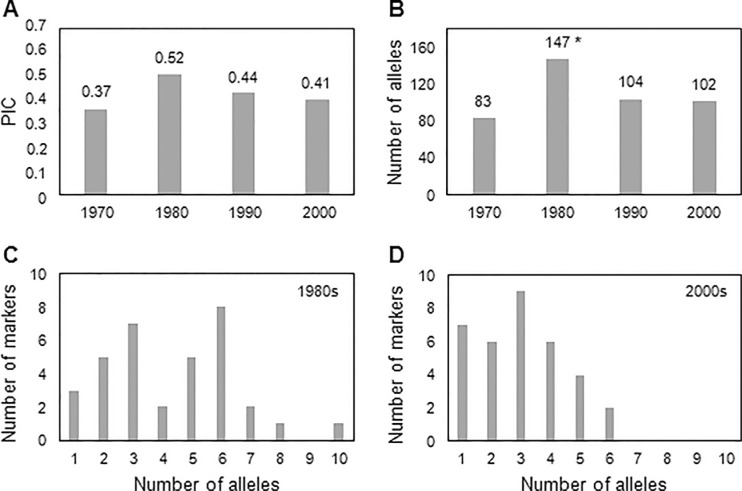
PIC values (A) and total number of alleles (B) of the genotypes developed in Brazil in the last four decades. Frequency of alleles per marker is shown for genotypes developed in the 1980s (C) and after 2000 (D). Number of genotypes per decade are: 5 (1970); 18 (1980); 8 (1990) and 16 (2000). An asterisk indicates statistical difference by t-test (p ≤ 0.05).

Furthermore, indicators of locus diversity (polymorphism information content - PIC) were calculated (Table 3). Large variation was observed among the polymorphic loci; PIC values ranged from 0.07 (EBmac0669) to 0.86 (Bmag120) with an average of 0.57. Not considering the monomorphic loci, only five markers originated PIC values below 0.50, therefore the majority of the markers were moderately to highly informative, according to the criteria proposed by Botstein *et al.* (1980). Among the five loci with PIC <0.50, three were located on chromosome four. Chromosomes four and six showed the lowest mean PIC value (0.43) among all chromosomes. Among the different sets, the highest PIC value was observed for wild barley (0.63) evidencing higher genetic diversity in these accessions. The second highest PIC value was found in the foreign genotypes (0.57), while PIC values varied from 0.27 (Companhia Antarctica Paulista) to 0.46 (CNPT-Embrapa) among the cultivars developed by the different breeding programs in Brazil. The PIC value considering all Brazilian genotypes (cultivars and breeding lines) was 0.51. It indicates a lower genetic diversity compared with foreign (0.57) and wild (0.63) accessions. Furthermore, the PIC value was lower among Brazilian genotypes developed in the last two decades in comparison to materials released in the 1980s (Figure 1A) suggesting a trend toward decreased diversity. The loss of genetic diversity is also supported by the statistically superior number of alleles detected in the genotypes developed in the 1980s (Figure 1B). Moreover, a change in the frequency of alleles was clearly observed between genotypes developed in the 1980s and 2000s where a reduced number of alleles per marker was observed in the genotypes developed in the 2000s (Figures 1C,D). The frequency of alleles per marker between genotypes from these two decades can be compared since a similar number of accessions developed in the 1980s and 2000s were analyzed (18 and 16, respectively).

### Genetic similarity

**Figure 2:**
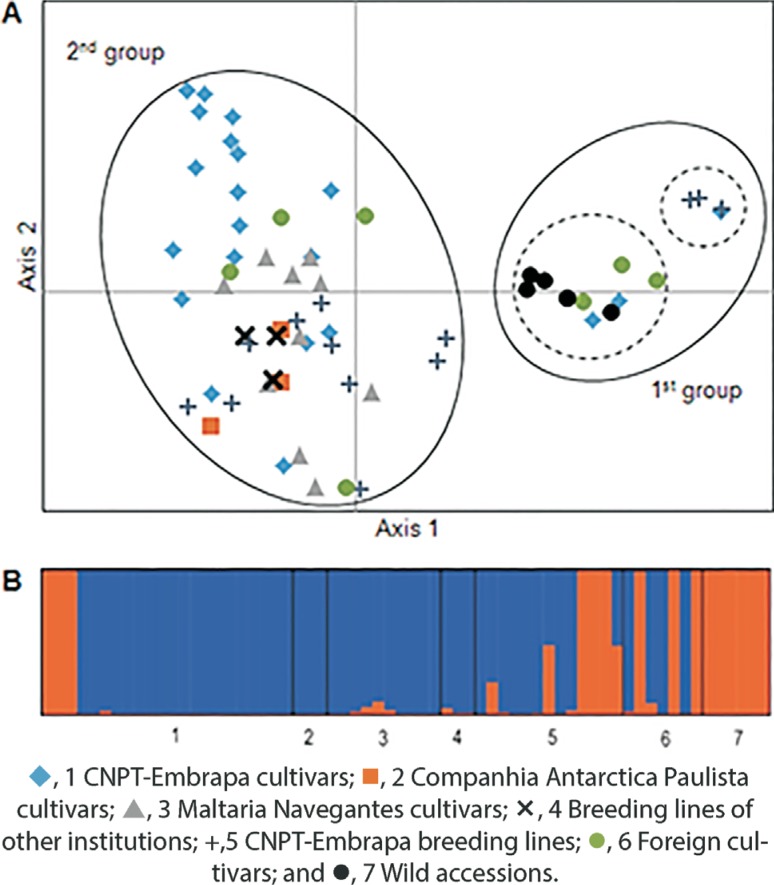
Analysis plots of the barley accessions based on the polymorphism of 34 microsatellite markers. (A) By Principal Coordinate Analysis (PCoA) two groups were identified, with the first one separated into two subgroups. Two symbols, in the first group, indicating wild genotypes (Hspo PI 466394 and Hspo PI 466396), and another two in the second group, indicating CNPT-Embrapa cultivars (Cevada BR2 and Embrapa 127), overlapped. (B) Estimated proportion of membership in the corresponding clusters (K = 2) as calculated using STRUCTURE software.

In order to assess the clustering of barley accessions based on SSR polymorphism, we conducted a Principal Coordinates Analysis (PCoA). The scatter plots for the two first axes showed that accessions formed two principal groups (Figure 2A). The first group (small circle on the right-hand side of the PCA plot) was further subdivided into two. One subgroup (larger dotted circle in Figure 2A) contained all wild accessions, two CNPT-Embrapa cultivars (Paraí and BRS 180), and three foreign cultivars (Dayton, Quest and Murasakimochi), while the other subgroup (smaller dotted circle in Figure 2A) included three CNPT-Embrapa breeding lines (PFC 88209, PFC 88210, and PFC 88211), and one CNPT-Embrapa cultivar (Vacaria). The second group (large circle on the left-hand side of the PCA plot) was composed of all other accessions and showed higher variation. Within this second group, the CNPT-Embrapa cultivars were scattered over a large area, with most of them being located in a separate region.

The AMOVA analysis revealed that 15% of the genetic variation (P <0.001) was distributed among the seven sets, representing the different breeding programs, countries and type of accession (wild/domesticated and breeding lines/cultivars), whereas 85% (P <0.001) was within the sets. When we consider only accessions developed in Brazil, the AMOVA showed 12% of the genetic variation among sets, and 88% (P <0.001) within sets (P <0.001). These results indicated that the genetic variation within the sets contributed to most of the genetic diversity detected.

**Figure 3:**
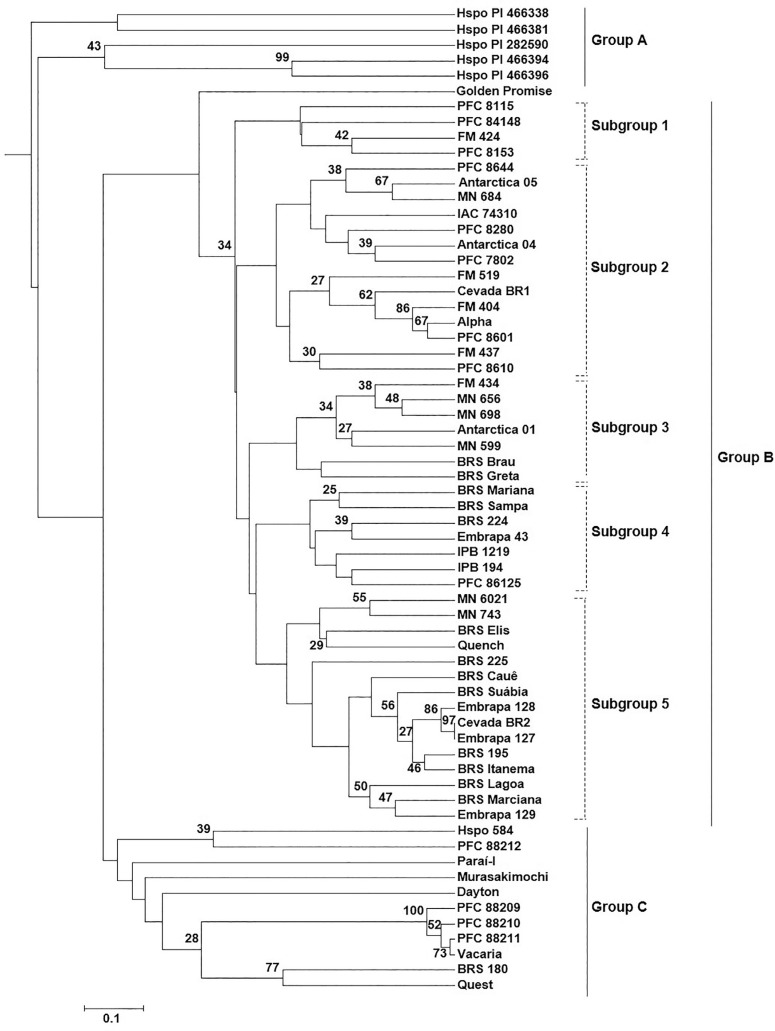
UPGMA tree of barley accessions constructed based on the matrix of shared microsatellite alleles. Bootstrap values are indicated at nodes. Only bootstrap values ≥ 25% are shown.

The UPGMA cluster analysis produced three main groups (named as Groups A, B and C) (Figure 3). Group A consisted of all wild accessions except Hspo 584, which was clustered in the Group C. Besides Hspo 584, Group C also contained 10 other accessions represented by breeding lines and cultivars developed by CNPT-Embrapa and foreign genotypes. When comparing the accessions present in Groups A and C with the ones belonging to the first group detected by the PCoA analysis, only the line PFC 88212 was different between them revealing agreement patterns across analyses.

Group B in the dendrogram was the largest, consisting of 48 accessions representing four foreign materials and 44 Brazilian genotypes. This means that 86% of the Brazilian genotypes were clustered inside the same group. Group B was further substructured into five subgroups. Subgroup one consisted of three CNPT-Embrapa breeding lines and one cultivar developed by Maltaria Navegantes. Subgroup two contained breeding lines developed by CNPTEmbrapa and Instituto Agronômico de Campinas (IAC), cultivars released by CNPT-Embrapa, Companhia Antarctica Paulista and Maltaria Navegantes, and one foreign genotype (Alpha). Subgroup three consisted of cultivars developed by the three breeding programs in Brazil, while subgroup four was represented by cultivars and breeding lines developed by CNPT-Embrapa and by other institution. Finally, subgroup five included twelve CNPT-Embrapa cultivars, one cultivar developed by Maltaria Navegantes and two foreign genotypes (MN 6021 and Quench). Interestingly, Golden Promise appeared separated within this Group B. When considering the different breeding programs in Brazil, the genotypes developed by CNPT-Embrapa were the only ones distributed across more than one group and across all the subgroups within Group B.

The Bayesian clustering results, implemented in STRUCTURE software, were in agreement with the results of the PCoA and UPGMA analyses. In this analysis we observed that the best inferred number of clusters was *K* = 2, obtained by the Evanno's method (Figure 2B). The majority of accessions of each group were grouped preferentially in one or the other cluster. Remarkably, the great majority of genotypes clustered in Group B by UPGMA analysis were assembled to the same group by Bayesian clustering. In addition, those clustering in Groups A and C were assigned to the other group. Exceptions were PFC 88212 and PFC 8610 that presented dual ancestry.

## Discussion

Brazil ranks third among the countries with the largest beer production in the world. However, it produces only around 300 thousand tons of barley per year, which results in one of the largest barley import needs (de Mori and Minella, 2012). Therefore, increments in barley production are essential. The demand for higher yielding and betteradapted crop varieties has increased the necessity to exploit the genetic variation of genebank collections (Keilwagen *et al.*, 2014). Evaluation of the genetic diversity of barley accessions can be an important source of information in order to discover and exploit novel alleles to be used in breeding programs. Here, for the first time, the SSR variability of Brazilian barley genotypes is reported. Although other groups have previously published molecular analyses of Brazilian barley accessions (Echart-Almeida and CavalliMolina, 2000; Kroth *et al.*, 2005; Maris AF, 1992, Bachelor's thesis, Universidade Federal do Rio Grande do Sul, Porto Alegre, RS), the number of genotypes evaluated in this report is higher and also the use of SSR markers is advantageous when compared to RAPD or isoenzymes used previously. SSR marker analysis have proven to be a method of choice for marker-assisted selection in breeding and genetic diversity studies (Varshney*et al.*, 2007), largely because they are highly informative and abundant in genomes, codominantly inherited and multiallelic (Gupta *et al.*, 1996; Oliveira *et al.*, 2006).

We successfully amplified 34 barley SSR barley loci obtaining an average of eight alleles for each locus. This is similar to the results reported for Iranian barley landraces (Khodayari *et al.*, 2012), higher than the one reported for Indian barley (Jaiswal *et al.*, 2010) and lower than the ones published by others (Saghai Maroof *et al.*, 1994;Malysheva-Otto *et al.*, 2006; Yahiaoui *et al.*, 2008; Nandha and Singh, 2014). Here we obtained a mean PIC value of 0.57, however, when considering just the Brazilian genotypes the diversity index was lower than that of foreign and wild accessions (Table 2), illustrating the low diversity in the Brazilian accessions. One interesting aspect is that in the UPGMA results (Figure 3) clusters seen inside Group B correlate with cultivar release date. Most of the accessions (77%) in subgroups one and two are older genotypes developed between 1970 and 1988, while 79% of the genotypes in the other subgroups were developed between 1989 and 2013. Only one genotype developed after the year 2000 (MN 684) was clustered outside of subgroups three, four and five and, among the 13 Brazilian genotypes in subgroup five, only three were developed before the year 2000. This means that cultivars released in the last years are more similar, indicating a decrease in the genetic diversity. Other indicators of the tendency to decreased genetic diversity are the lower number of alleles, the lower frequency of alleles per marker, and lower PIC values obtained for Brazilian genotypes developed after the 1980s (Figure 1).

The observation that Brazilian barleys are closely related is in agreement with other studies (Selbach and Cavalli-Molina, 2000;Kroth *et al.*, 2005). In fact, crop breeding programs have drastically narrowed the genetic diversity in cultivated plants, reducing tolerance to environmental stresses (Nevo, 2007). This genetic diversity reduction may have occurred because of the selection of specific alleles that confer stability in the specific environment of Southern Brazil. An evidence of the use of common materials for obtaining the cultivars can be visualized by the analysis of Group B in the dendrogram (Figure 3). The genotypes Volla and Wpgm 626-46-25 are present in the genealogy of three accessions in subgroup one (Table 1). Alpha is an ancestor of Antarctica 04, FM 519, Cevada BR1, FM 404, and FM 437, which clustered in subgroup two (Table 1). In subgroup four, most of the accessions were developed by CNPT-Embrapa. The genotypes BRS Mariana and BRS Sampa, which were closely related, share PFC 9205 in their genealogy. The genotype IPB 194 was used in the cross that generated Embrapa 43, which was then used to obtain BRS 224 (Table 1). Most of the genotypes grouping in subgroup five have BRS 195 and Cevada BR2 in their genealogy (Table 1). Hence, barley breeding programs in Brazil should definitely consider the use of a more diverse set of materials in their crossings.

The UPGMA results (Figure 3) were in agreement with PCoA and STRUCTURE analysis (Figure 2). The three groups detected in the UPGMA plot showed a pattern correlated to the number of rows of grains on the ear and to barley type (wild or domesticated). For instance, Group A was exclusively formed by wild accessions (two-row genotypes), while all genotypes in Group C, except for the wild accession Hspo 584, were representatives of six-row barley. These results could suggest that Hspo 584 has a different geographical origin than the other wild accessions. Although it has been shown that SSR and SNP markers can clearly separate the two- and six-rowed barley groups (Hayes and Szucs, 2006; Khodayari *et al.*, 2012), exceptions have been reported where a few two- and six-rowed genotypes showed similarity (Marmiroli *et al.*, 1999; Brantestam *et al.*, 2007; Lamara *et al.*, 2013), as we also found here.

Wild barley (*H. vulgare* ssp. *spontaneum*), the progenitor of cultivated barley, is a selfing annual grass distributed over a wide ecological range that differs in water availability, temperature, soil type, altitude and vegetation, thus generating a high potential for adaptive genetic diversity against abiotic and biotic stresses. It has been reported that wild barley has developed unique mechanisms for surviving harsh environments, mainly through developing new genetic variations and alleles (Nevo and Chen, 2010). Here we detected a greater diversity in wild barley accessions when compared to domesticated ones. Higher numbers of SSR alleles and greater diversity levels of individual loci were also found for wild genotypes in comparison to cultivated barley (Saghai Maroof*et al.*, 1994; Nandha and Singh, 2014). Another evidence for greater genetic diversity in wild barley is the higher number of private alleles detected (Table S2), a result that has also been reported byNandha and Singh (2014). Interestingly, cultivars Vacaria, Paraí, and BRS 180 and breeding lines PFC 88209, PFC 88210, and PFC 88211 developed by CNPT-Embrapa, as well as three foreign cultivars (Dayton, Quest, and Murasakimochi) grouped together with one wild accession (Hspo 584) in all analyses performed here. The genetic diversity shared by these accessions and wild barley indicates the potential of these accessions as a source of alleles for breeding purposes. A close similarity between domesticated barley and one wild accession has also been reported by Nandha and Singh (2014).

In this study, the geographic origin was not clearly associated with the groups, despite previous observation that SSR markers can differentiate barleys according to their region of origin (Malysheva-Otto *et al.*, 2006). However, other associations can be done based on genotype similarity. For instance, Group C consisted of six accessions that are feed varieties (Paraí-I, PFC 88209, PFC 88210, PFC 88211, PFC 88212, and Vacaria), whereas all accessions within Group B are malting barleys.

The highest average PIC was found for chromosome three, the same one where the highest diversity parameters were detected by Malysheva-Otto *et al.* (2006). Chromosome four was the one with the highest number of markers evaluated, however it showed the lowest PIC values. Among these loci, one was monomorphic and the others had PIC values below 0.50. Interestingly, chromosome four contains the *HvAACT1* gene responsible for the aluminum tolerance in barley (Furukawa *et al.*, 2007; Wang *et al.*, 2007). Aluminum is an important constraint in Southern Brazil (Echart and Cavalli-Molina, 2001), where most of Brazilian barley is produced. It has been proposed that the low variability for aluminum tolerance in Brazilian barley indicates that significant increments through conventional breeding are unlikely (Minella, 2001). It is possible that the low variability found in chromosome four could explain the low level of aluminum tolerance diversity in Brazilian barley. Strategies must be considered to surpass the low variability for some traits in barley, allowing the increase in stress tolerance and, ultimately, yield.

In conclusion, we have detected a lower PIC value among barley genotypes bred in Brazil compared with foreign and wild genotypes. A tendency to a decrease in genetic diversity of Brazilian barley is occurring with modern materials released over the last two decades, showing lower number of alleles, lower frequency of alleles per marker, and lower PIC values in comparison to cultivars released in the 1980s. This could be partially explained by the use of common ancestors carrying important traits. The use of more diverse material could be of interest to exploit novel alleles in crop breeding programs without losing quality. Some of the genotypes used here, such as the ones present in Group C of the dendrogram, should be considered. Private alleles unique to Brazilian genotypes are important for future use in cultivar identification.

## Supplementary Material

The following online material is available for this article:

Table S1 - Characteristics of the SSR markers.

Table S2 - Private alleles.

This material is available as part of the online article from http://www.scielo.br/gmb
