# Supplementary material for: Assessment of genetic diversity in Brazilian barley using SSR markers
Source: Genet Mol Biol. 2016 Jan-Mar;39(1):86–96. doi: 10.1590/1678-4685-GMB-2015-0148 (PMC4807376; doi:10.1590/1678-4685-GMB-2015-0148)
Supplement: Supplementary file 1 [file 1415-4757-gmb-39-1-86-Suppl01.pdf]

Table S1 Characteristics of the SSR markers used. Forward or reverse primer contains a M13 sequence at the 5' end. See Materials and Methods for details about the PCR program and reaction mix.

Marker	Forward sequence	Reverse sequence	Chromosome	Dye	PCR Program[2]	Mix
Bmac0090	ACATCAACCCTCCTGCTC	CCGCACATAGTGGTTACATC	1H	PET	TD60-55	D
Bmag0032	CCATCAAAGTCCGGCTAG	GTCGGGCCTCATACTGAC	1H	NED	TD60-55	A
Bmag211	ATTCATCGATCTTGTATTAGTCC	ACATCATGTCGATCAAAGC	1H	VIC	TD60-55	A
HVM20	CTCCACGAATCTCTGCACAA	CACCGCCTCCTCTTTCAC	1H	FAM	TD60-55	C
Bmag0125	AATTAGCGAGAACAAAATCAC	AGATAACGATGCACCACC	2H	PET	TD60-55	D
Bmag0378	CTTTTGTTTCCGTAGCATCTA	ATCCAACTATAGTAGCAAAGCC	2H	VIC	TD60-55	C
Bmag0749	CGGATTCTTGAGTAGTCTCTG	GATCTGTTTTTGTAGAACATGC	2H	FAM	TD60-55	C
HVM36	TCCAGCCGACAATTTCTTG	AGTACTCCGACACCACGTCC	2H	PET	TD60-50	A
HVM54	AACCCAGTAACACCTGTCCTG	AGTTCCCTGACCCGATGTC	2H	PET	TD60-55	C
Bmac0067	AACGTACGAGCTCTTTTTCTA	ATGCCAACTGCTTGTTTAG	3H	VIC	TD60-55	A
Bmag0013	AAGGGGAATCAAAATGGGAG	TCGAATAGGTCTCCGAAGAAA	3H	FAM	TD60-55	D
Bmag225	AACACACCAAAAATATTACATCA	CGAGTAGTTCCCATGTGAC	3H	VIC	TD60-50	C
HVM60	CAATGATGCGGTGAACTTTG	CCTCGGATCTATGGGTCCTT	3H	FAM	TD60-50	C
Bmac0030	CCCAATCGGAGTTACAGATG	GCCTCTCTGAGAATGGATC	4H	NED	TD60-55	C
Bmac0310[1]	CTACCTCTGAGATATCATGCC	ATCTAGTGTGTGTTGCTTCCT	4H	NED	TD60-55	A
Bmag0353[1]	ACTAGTACCCACTATGCACGA	ACGTTCATTAAAATCACAACTG	4H	FAM	TD60-55	C
EBmac0669	TCTACACTACACATGTTATCTTGC	GTGTTGATCTTCCTCCTAGTG	4H	NED	TD60-50	D
EBmag0781	CTATTTTCTAATGCTTGGACC	TGTCTAGTTCATCATCATTGC	4H	NED	TD60-55	C
HVM68[1]	AGGACCGGATGTTCATAACG	CAAATCTTCCAGCGAGGCT	4H	PET	TD60-55	D
HvML03	CTTCCATGTCACCTACAG	CGAACTGGTATTCCAAGG	4H	VIC	TD60-50	C
wms165	TGCAGTGGTCAGATGTTTCC	CTTTTCTTTCAGATTGCGCC	4H	NED	TD60-55	D
XGWM6	CGTATCACCTCCTAGCTAAACTAG	AGCCTTATCATGACCCTACCTT	4H	FAM	TD60-55	C
Bmac0113	TCAAAAGCCGGTCTAATGCT	GTGCAAAGAAAATGCACAGATAG	5H	PET	TD60-55	D
Bmac096	GCTATGGCGTACTATGTATGGTTG	TCACGATGAGGTATGATCAAAGA	5H	NED	TD60-55	A
Bmag0387	CGATGACCATTGTATTGAAG	CTCATGTTGATGTGTGGTTAG	5H	VIC	TD60-55	C
HvLOX	CAGCATATCCATCTGATCTG	CACCCTTATTTATTGCCTTAA	5H	VIC	TD60-55	D
Bmac0316	ATGGTAGAGGTCCCAACTG	ATCACTGCTGTGCCTAGC	6H	NED	TD60-55	A
Bmac251	CAGATCTCAACAAACACACC	ATTCACTATAAACATATGGGTCC	6H	PET	TD60-50	D
Bmag0173	CATTTTTGTTGGTGACGG	ATAATGGCGGGAGAGACA	6H	PET	TD60-55	C
HVM65	AGACATCCAAAAAATGAACCA	TGGTAACTTGTCCCCCAAAG	6H	FAM	TD60-55	D
Bmac0031[1]	AGAGAAAGAGAAATGTCACCA	ATACATCCATGTGAGGGC	7H	NED	TD60-55	A
Bmac0167[1]	CATTTCCACTTCAAAATATCC	CCAAAGTTTGAGTGCAGAC	7H	PET	TD60-55	C

Bmag0120	ATTTCATCCCAAAGGAGAC	GTCACATAGACAGTTGTCTTCC	7H	NED	TD60-55	D
Bmag0135	ACGAAAGAGTTACAACGGATA	GTTTACCACAGATCTACAGGTG	7H	FAM	TD60-55	A

[1] Reverse primer contains a M13 sequence (TGTAAAACGACGGCCAGT) at the 5’ end.
[2] The PCR program contains a touchdown step (see Materials and Methods).
