# Supplementary material for: Assessment of genetic diversity in Brazilian barley using SSR markers
Source: Genet Mol Biol. 2016 Jan-Mar;39(1):86–96. doi: 10.1590/1678-4685-GMB-2015-0148 (PMC4807376; doi:10.1590/1678-4685-GMB-2015-0148)
Supplement: Supplementary file 2 [file 1415-4757-gmb-39-1-86-Suppl02.pdf]

Table S2 Private alleles detected represented accordingly to institution, country, barley type and marker.

	CNPT/Embrapa cultivars	Companhia Antarctica Paulista	Maltaria Navegantes	Breeding lines from other institutions	Breeding lines from CNPT/Embrapa	Foreign genotypes	Wild barley
Bmac0090	0	0	0	0	0	0	2
Bmag0211	0	0	0	0	0	1	2
Bmag0032	3	0	1	0	1	2	5
HVM20	0	0	0	0	0	0	2
Bmag0125	0	0	0	0	1	1	1
Bmag0378	0	0	0	0	0	1	4
Bmag0749	0	0	0	0	0	0	1
HVM36	0	0	0	0	0	1	1
HVM54	0	0	1	0	0	2	1
Bmac0067	0	0	0	0	0	1	2
Bmag0013	0	0	1	0	0	1	4
Bmag0225	1	0	1	0	0	0	1
HVM60	1	0	0	0	0	1	3
Bmac0030	1	0	0	0	0	1	2
Bmac0310	1	0	0	0	0	2	2
Bmag0353	0	0	0	0	0	1	5
EBmac0669	0	0	0	0	0	0	0
EBmag0781	0	0	0	0	0	0	1
HVM68	1	0	0	0	0	2	3
HvML03	0	0	0	0	1	2	2
wms165	0	0	0	0	1	2	2
XGWM6	0	0	0	0	0	0	0
Bmac0113	1	0	0	0	0	1	2
Bmac0096	0	0	0	0	0	0	4
Bmag0387	2	0	0	0	0	0	1
HvLOX	0	0	0	0	0	0	0
Bmac0316	3	0	0	1	1	0	6
Bmac0251	0	0	0	0	0	0	0
Bmag0173	0	0	0	0	1	0	1
HVM65	0	0	0	0	0	0	0
Bmac0031	1	0	0	0	0	1	6
Bmac0167	4	0	0	0	0	0	1

Bmag0120	1	0	1	0	2	0	2
Bmag0135	1	0	0	0	1	0	0
Total	21	0	5	1	9	23	69
